# Long-Term Pharmacological Inhibition of the Activity of All NOS Isoforms Rather Than Genetic Knock-Out of Endothelial NOS Leads to Impaired Spatial Learning and Memory in C57BL/6 Mice

**DOI:** 10.3390/biomedicines9121905

**Published:** 2021-12-14

**Authors:** Jhana O. Hendrickx, Sofie De Moudt, Elke Calus, Peter Paul De Deyn, Debby Van Dam, Guido R. Y. De Meyer

**Affiliations:** 1 Laboratory of Physiopharmacology, Faculty of Pharmaceutical, Biomedical and Veterinary Sciences, University of Antwerp, Universiteitsplein 1, 2610 Wilrijk, Belgium; jhana.hendrickx@uantwerpen.be (J.O.H.); sofie.demoudt@uantwerpen.be (S.D.M.); 2 Laboratory of Neurochemistry and Behaviour, Institute Born-Bunge, Department of Biomedical Sciences, University of Antwerp, Universiteitsplein 1, 2610 Wilrijk, Belgium; elke.calus@uantwerpen.be (E.C.); peter.dedeyn@uantwerpen.be (P.P.D.D.); debby.vandam@uantwerpen.be (D.V.D.); 3 Department of Neurology and Alzheimer Research Center, University of Groningen and University Medical Center Groningen, Hanzeplein 1, 9713 GZ Groningen, The Netherlands

**Keywords:** arterial stiffness, cognitive decline, nitric oxide synthase

## Abstract

Increasing epidemiological and experimental evidence points to a link between arterial stiffness and rapid cognitive decline. However, the underlying mechanism linking the two diseases is still unknown. The importance of nitric oxide synthases in both diseases is well-defined. In this study, we introduced arterial stiffness in both genetic (eNOS^−/−^, endothelial nitric oxide synthase knockout) and pharmacological (N(G)-nitro-L-arginine methyl ester (L-NAME) treatment) NO dysfunction models to study their association with cognitive decline. Our findings demonstrate that the non-selective inhibition of NOS activity with L-NAME induces cardiac dysfunction, arterial stiffness, and a decline in hippocampal-dependent learning and memory. This outcome demonstrates the importance of neuronal NOS (nNOS) in both cardiovascular and neurological pathophysiology and its potential contribution in the convergence between arterial stiffness and cognitive decline.

## 1. Introduction

In recent years, there has been increasing interest in the potential interaction between cardiovascular disease (CVD) and neurodegenerative syndromes. In the past, CVD and neurodegeneration were considered separate entities based on the clinical classification criteria. However, epidemiological studies increasingly report an independent, mechanistic convergence between both pathological syndromes. In recent decades, there has been increasing interest in the role of arterial stiffness in neurodegeneration, as it was found to be an independent risk factor for both CVD and neurodegeneration [1,2,3,4]. Although overwhelming evidence demonstrates an association between arterial stiffness and neurodegeneration in human subjects, these studies are limited by correlative evidence.

Although nitric oxide (NO) is a small gaseous signaling molecule, it has a well-defined importance in not only arterial stiffness [4,5,6], but also in neurodegeneration [7,8,9,10]. Studies in murine models of NO dysfunction through the inhibition of nitric oxide synthase (NOS) with N(G)-nitro-L-arginine methyl ester (L-NAME) or by a genetic knockout of endothelial NOS (eNOS^−/−^) [11,12], have shown the importance of NO in arterial stiffness. Additional studies using the same animal models have also demonstrated the importance of NO in neurodegeneration following the acquisition of cerebrovascular structural changes and cerebral infarcts as a result of its inhibition [13,14,15].

There are three NOS isoforms: neuronal NOS (nNOS), inducible NOS (iNOS), and endothelial NOS (eNOS), depending on their localization of action: nNOS is expressed in neurons, eNOS is expressed in endothelial cells, and iNOS is expressed in glia cells upon brain injury or inflammation [7,9]. Although for many years their functions were thought to be tissue-specific, increasing evidence suggests that they have a broad spectrum of physiological functions throughout the body.

In the present study, we sought to investigate in-depth the effect of arterial stiffness on the progression of neurodegeneration in both a well-known genetic eNOS dysfunction (eNOS^−/−^) mouse model and a pharmacological, non-selective NOS dysfunction mouse model (L-NAME treatment) of arterial stiffness.

## 2. Materials and Methods

### 2.1. Experimental Animals and Tissue Collection

Non-selective nitric oxide synthase inhibition was induced by pharmacological treatment of male C57BL/6 mice (The Jackson Laboratory, Bar Harbor, ME, USA) from the age of 8 weeks onward with 0.5 mg/mL N(G)-nitro-L-arginine methyl ester (L-NAME) in drinking water for 2 weeks (control *n* = 9; treated *n* = 10), 8 weeks (control *n* = 11; treated *n* = 11), and 16 weeks (control *n* = 10; treated *n* = 7). Compared to other studies [16,17], a lower L-NAME dosage was administered in order to obtain a partial inhibition of endothelial nitric oxide synthase (eNOS) activity in the aortic vessel wall.

In parallel, male eNOS knockout mice (eNOS^−/−^, The Jackson Laboratory, Bar Harbor, ME, USA) with a C57BL/6 background were investigated at 2 months (C57BL/6 control *n* = 14; eNOS^−/−^ *n* = 10), 4 months (C57BL/6 control *n* = 11; eNOS^−/−^ *n* = 10), and 6 months (C57BL/6 *n* = 25; eNOS^−/−^ *n* = 22) of age. All mice used in this study were bred and housed in the laboratory animal facility of the University of Antwerp. All mice were socially housed in standard mouse cages with a maximum of eight animals per cage under conventional laboratory conditions, with a constant room temperature (22 ± 2 °C) and humidity (55% ± 5%), and an artificial day/night cycle of 12 h/12 h (lights on at 8:00 a.m.). Food and water were provided ad libitum. L-NAME intake was assessed by weighing the drinking bottle at the beginning and end of each week, after which the L-NAME drinking water was refreshed. The average intake of L-NAME-treated mice was 3.3, 2.9, and 3.3 mg/day per animal at 2, 8, and 16 weeks of treatment, respectively. Experiments were approved by the Animal Ethics Committee of the University of Antwerp (ECD approval No. 2017/53, approved on 26 July 2017) and were carried out in accordance with the U.K. Animals (Scientific Procedures) Act, 1986 and associated guidelines, EU Directive 2010/63/EU for animals, and were performed in accordance with the Animal Research: Reporting of In Vivo Experiments (ARRIVE) Guidelines [18].

In the first week of the experiment, the spatial learning and memory of animals were assessed using the Morris water maze (MWM) test. During the second week, blood pressure measurements and echocardiographic analyses were performed, after which the mice were humanely killed under deep anesthesia (sodium pentobarbital (Sanofi, Belgium), 250 mg/kg, i.p. [19]) by perforation of the diaphragm, for further ex vivo determination of arterial stiffness.

### 2.2. Spatial Learning and Memory

Spatial learning and memory functions were evaluated at the age of 6 months (C57BL/6 vs. eNOS^−/−^ at the age of 6 months and untreated C57BL/6 vs. treated C57BL/6 animals after 16 weeks of treatment) by means of the MWM test [20,21]. The MWM consisted of a circular pool (diameter: 150 cm, height: 30 cm) filled with opacified water using non-toxic white paint and kept at 25 °C. Invariable visual cues were placed around the pool. The MWM consisted of an acquisition phase and a probe trial. The acquisition phase was performed over a period of 4 days and consisted of 2 daily trial blocks (1 at 10:30 a.m. and 1 at 03:00 p.m.) of 4 trials with a 15 min inter-trial interval. During the acquisition phase, a round acrylic glass platform (diameter 15 cm) was placed 1 cm below the water surface on a fixed position in the center of one of the pool’s quadrants. Mice were placed in the water facing the wall and were recorded while trying to find the hidden platform for a maximum duration of 120 s. If the mouse was not able to reach the platform within 120 s, it was guided to the platform, where they had to stay for 15 s before being returned to their home cage. The starting positions varied in a semi-random order. The probe trial followed 4 days after the final acquisition trial. For this trial, the platform was removed, mice were placed in the MWM at a fixed position, and swimming trajectories were recorded for a period of 100 s. During both acquisition and probe trials, the animals’ trajectories were recorded using a computerized video-tracking system (Ethovision, Noldus, The Netherlands), with path length, escape latency, and swimming speed recorded. Spatial accuracy is expressed as percentage of time spent in each quadrant of the MWM, i.e., the specific location of the platform during the acquisition phase. The experimenters were blind to the genetic or treatment status of all mice.

### 2.3. Blood Pressure Measurements

Peripheral blood pressure was measured using a non-invasive CODA tail-cuff blood pressure system (KENT Scientific CO., Torrington, CT, USA), as previously described [22]. Mice were immobilized in a Plexiglas restrainer and an occlusion cuff and volume pressure cuff were placed around the tail of the mouse. Voltage output from both cuffs were recorded and analyzed by PowerLab signal transduction unit associated chart software (AD Instruments). To minimize discomfort for the animals and to increase the reliability of measurements, the blood pressure of the animals was measured 3 days prior to the effective measurement on day 4. For each mouse, a measurement consisted of 15 cycles (approximately 15 min per animal) and the reported values are the averaged values measured on day 4.

### 2.4. Echocardiography

Echocardiography was performed with a high-frequency, high-resolution digital imaging platform with linear array technology and color Doppler mode for in vivo micro-imaging (Vevo^®^ 2100 Imaging System, FUJIFILM Visual Sonics Inc., Toronto, ON, Canada). To assess systolic and diastolic heart function in mice, a high-frequency transducer probe (Visual Sonics MS500D, FUJIFILM Visual Sonics, Inc., Toronto, Canada with a frequency range of 18–38 MHz) was used to provide the appropriate resolution and depth of penetration needed. Transthoracic echocardiograms were performed on anesthetized mice (97% O_2_ and 3% isoflurane (Forene, Abbvie, North Chicago, IL, USA) for induction and 98.5% O_2_ and 1.5% isoflurane for maintenance). Mice were placed on a preheated platform in a supine position in order to maintain their body temperature at 36–38 °C. Actual body temperatures were measured by means of an anal thermometer probe and were continuously monitored throughout the whole procedure. Isoflurane concentrations were titrated (1–2%) during imaging to maintain heart rate at 500 ± 50 beats/min. Systolic left ventricular dimensions were acquired via short-axis M-mode images. To calculate the percentage of fractional shortening (FS%) and ejection fraction (EF%), end-systolic and end-diastolic dimensions along with end-systolic and end-diastolic volumes and stroke volume were recorded via short-axis M-mode images. Diastolic cardiac function was determined using color and pulse wave (PW) Doppler recordings of the trans-tricuspid flow. Reported cardiac parameters consist of averaged measurements of three consecutive M-mode and/or PW Doppler images.

### 2.5. Non-Invasive Pulse Wave Velocity (PWV) Measurements of the Aortic Abdominal Aorta (aPWV)

A high-frequency, high-resolution digital imaging platform (Vevo^®^ 2100 Imaging System, FUJIFILM Visual Sonics Inc., Toronto, ON, Canada) was used on anesthetized mice (isoflurane in O_2_; 3% for induction and 1.5% for maintenance; Forene, Abbvie, North Chicago, IL, USA)) to assess pulse wave velocity measurements of the abdominal aorta (aPWV). Body temperature was maintained at 36–38 °C and mice were continuously monitored and isoflurane concentrations were titrated (1–2%) during imaging to maintain heart rates at 500 ± 50 beats/min (bpm). PWV measurements were performed with a 24 MHz transducer (Visual Sonics MS400, FUJIFILM Visual Sonics, Inc., Toronto, ON, Canada) using the method developed by Di Lascio et al. [23]. In brief, a 24 MHz transducer was placed on the abdomen of the animal and B-mode images of 700 frames per second of the abdominal aorta and carotid artery were obtained using the EKV imaging mode to measure aortic diameter (D). A PW Doppler tracing was obtained to measure aortic flow velocity (V). Velocity was plotted against the natural logarithm of the diameter, and the slope of the linear part of the resulting ln(D)-V loop was used to calculate PWV values using MATLAB v2014 (Mathworks, Natick, MA, USA).

### 2.6. Rodent Oscillatory Tension Set-Up for Arterial Compliance (ROTSAC)

After humanely killing the animals, the thoracic aorta was carefully removed and cleared of adherent tissue. Starting approximately two millimeters distal to the aortic arch, the descending thoracic aorta was cut into four segments of two millimeters’ length for further vascular reactivity and stiffness analyses. These segments were immediately immersed in Krebs Ringer (KR) solution (37 °C, 95% O_2_/5% CO_2_, pH 7.4) containing: NaCl (118 mmol/L), KCl (4.7 mmol/L), CaCl_2_ (2.5 mmol/L), KH_2_PO_4_ (1.2 mmol/L), MgSO_4_ (1.2 mmol/L), NaHCO_3_ (25 mmol/L), CaEDTA (0.025 mmol/L), and glucose (11.1 mmol/L). The KR solution was continuously aerated with a 95% O_2_/5% CO_2_ gas mixture to maintain the pH at 7.4 and was replaced periodically to prevent glucose depletion. Aortic segments were mounted between two parallel wire hooks in 10 mL organ baths filled with KR solution (37 °C, 95% O_2_/5% CO_2_, pH 7.4). The diameter and estimates of transmural pressure were derived as previously described [24]. In short, the force and displacement of the upper hook were measured with a force-length transducer connected to a data acquisition system (Powerlab 8/ 30 and LabChart Pro, AD Instruments Inc., Colorado Spring, CO, USA). Force and displacement were acquired at 0.4 kHz. To estimate the transmural pressure that would exist in the equilibrated vessel segment with the given distension force and dimensions, the Laplace relationship was used. All measurements were performed over a pressure range with pressure clamps between diastolic 80 to systolic 120 mm Hg at 10 Hz was chosen to allow calculation of the Peterson modulus (Ep):

(1) $$\mathrm{Ep}=D_{0}\cdot \frac{\Delta P}{\Delta D}$$

where ∆D is the difference between systolic and diastolic diameter, ∆P is the pressure difference of 40 mm Hg, and D_0_ is the diastolic diameter. This pulse pressure difference of 40 mmHg, applied at a frequency of 10 Hz, and corresponding to a physiological heart rate of 600 beats per minute in mice, was kept constant throughout the experiment. The measurements took 5–10 min on average.

### 2.7. Statistical Analysis

Data are presented as mean ± SEM unless otherwise indicated. A factorial ANOVA was performed with genotype, treatment, and time as factors. Differences between genotypes/treatments were considered significant at *p* < 0.05. Applied statistical analyses are shown in the figure legends and were performed using GraphPad Prism (version 9.1.2 for Windows, GraphPad Software, San Diego, CA, USA). MWM probe trial results were additionally analyzed with Dirichlet distributions as described earlier [25], using R language programming in Jupyter Notebook [26] with the Dirichlet package from Eric Suh (Fitting the parameters of a Dirichlet distribution), available at: https://github.com/ericsuh/dirichlet (accessed on 13 December 2021).

## 3. Results

### 3.1. Chronic L-NAME Treatment Induces Hypertrophic Cardiomyopathy

**Figure 1:**
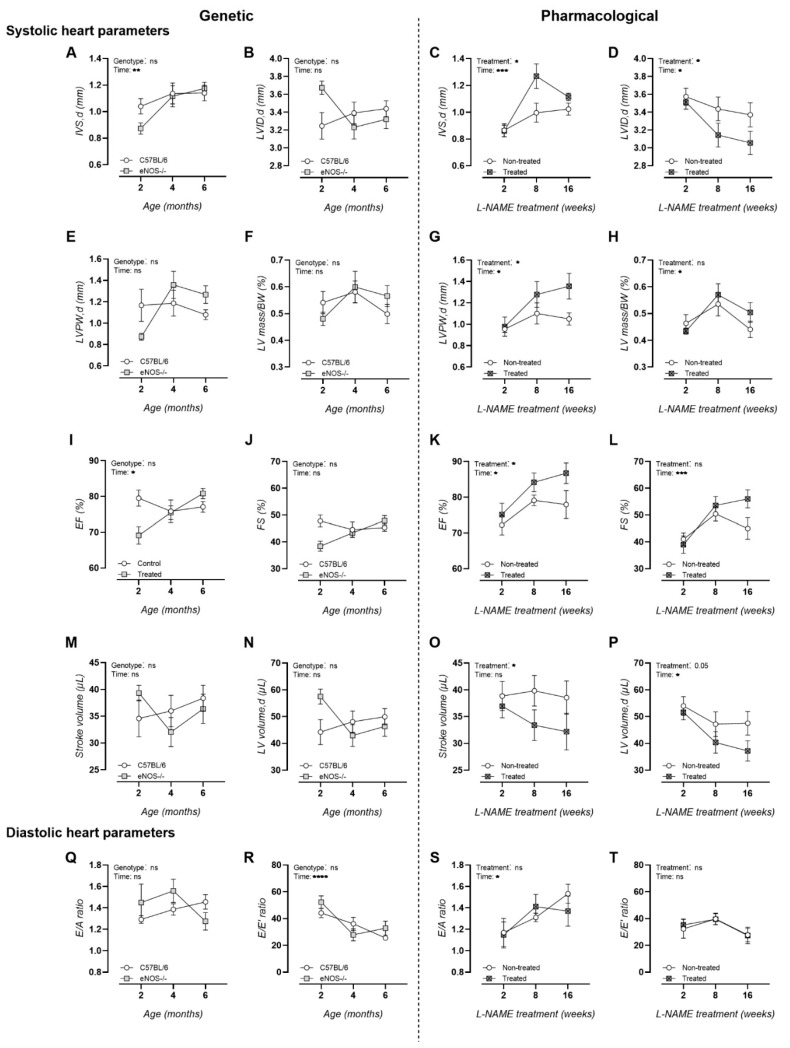
Systolic and diastolic echocardiographic heart function analysis of studied murine models. Results of the genetic NO dysfunction murine model are depicted in the left model and those of the pharmacologically induced NO dysfunction murine model in the right one. Systolic heart parameters: (**A**,**C**) IVS,d; (**B**,**D**) LVID,d; (**E**,**G**) LVPW,d; (**F**,**H**) LVmass/BW; (**I**,**K**) EF; (**J**,**L**) FS; (**M**,**O**) stroke volume; and (**N**,**P**) LV volume,d. Diastolic heart parameters: (**Q**,**S**) E/A and (**R**,**T**) E/E′. Factorial ANOVA for the factors genotype and treatment; * *p* < 0.05, ** *p* < 0.02, *** *p* < 0.002, **** *p* < 0.0002. Data are presented as mean ± SEM. IVS,d = inner ventricular septum thickness during diastole; LVID,d = left ventricular inner diameter during diastole; LVPW,d = left ventricular posterior wall thickness during diastole; LV mass/BW = left ventricular mass corrected for body weight; EF = ejection fraction; FS = fractional shortening; E/A = peak velocity blood flow in early diastole (**E**) to peak velocity flow in late diastole (**A**); E/E′ = mitral peak velocity of early filling (**E**) to early diastolic mitral annular velocity (E′).

In contrast to eNOS^−/−^ mice, echocardiography revealed signs of hypertrophy in L-NAME-treated animals by means of a significantly increased inner-ventricular septum thickness (Figure 1A,C) and left-ventricular posterior wall thickness (Figure 1E,G), alongside a significantly decreased left-ventricular inner-diameter (Figure 1B,D) and stroke volume (Figure 1M,O) and a trend toward decreased left ventricular ejection volumes during diastole (Figure 1N,P). In addition, ejection fractions were significantly elevated upon L-NAME treatment (Figure 1I,K), indicative of hypertrophic cardiomyopathy (Figure 1B,D). Other measurements of systolic heart functionality, i.e., left ventricular mass corrected for body weight (Figure 1F,H) and fractional shortening (Figure 1J,L), as well as measurements of diastolic heart functionality, remained unchanged in both murine models (Figure 1Q–T).

### 3.2. NO Dysfunction Is Associated with Hypertension

**Figure 2:**
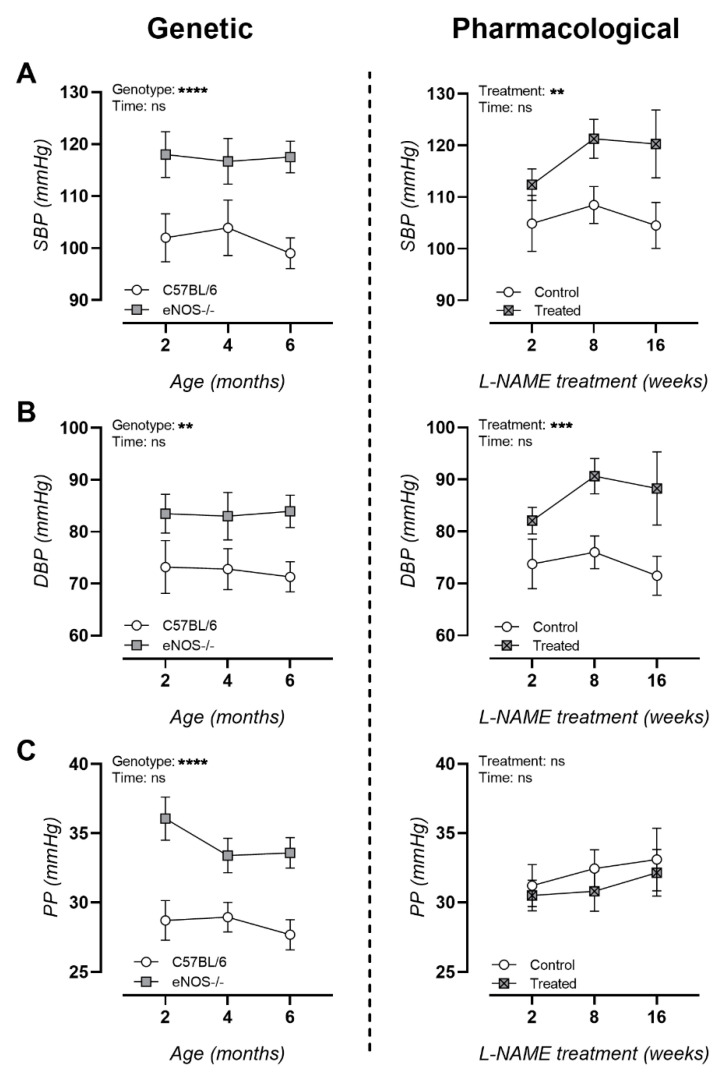
Longitudinal peripheral blood pressure measurements in a genetic and pharmacologically manipulated murine models of NO dysfunction. (**A**) Systolic blood pressure (SBP); (**B**) diastolic blood pressure (DBP) and (**C**) pulse pressure (PP) for genetic (eNOS^−/−^, left column) and pharmacologically treated (L-NAME, right column) animals. Factorial ANOVA for the factors of genotype/treatment and time; ** *p* < 0.02, *** *p* < 0.002, **** *p* < 0.0002. Data are presented as mean ± SEM.

Peripheral blood pressure measurements showed significantly increased systolic and diastolic blood pressure in both murine models (Figure 2A,B). In contrast to L-NAME-treated animals, eNOS^−/−^ animals presented chronically elevated pulse pressures (Figure 2C).

### 3.3. NO Dysfunction Is Characterized with Arterial Stiffness In Vivo and Ex Vivo

**Figure 3:**
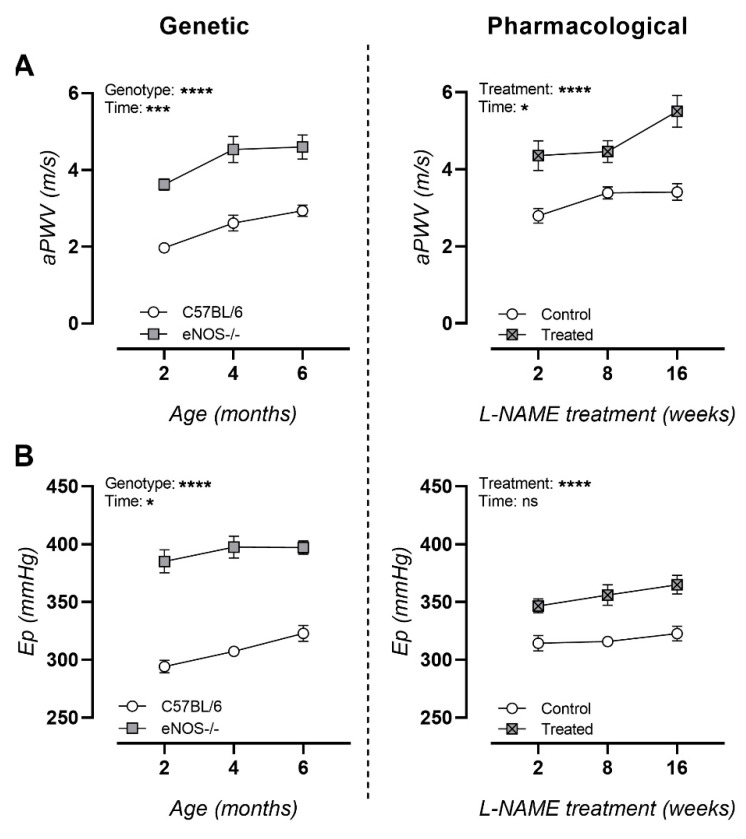
Longitudinal arterial stiffness measurements in genetic and pharmacologically manipulated murine models of NO dysfunction. (**A**) In vivo measurements of aPWV and (**B**) ex vivo measurements of elastic Peterson modulus (Ep) for genetic (eNOS^−/−^, left column) and pharmacologically treated (L-NAME, right column) animals. Factorial ANOVA for the factors genotype/treatment and time; * *p* < 0.05, *** *p* < 0.002, **** *p* < 0.0002. Data are presented as mean ± SEM.

An in vivo assessment of arterial stiffness resulted in marked age-dependent increases in aPWV in both murine models at all ages (Figure 3A). An ex vivo assessment of arterial stiffness revealed chronically elevated Ep values in both murine models, though more pronounced in eNOS^−/−^ mice (Figure 3B).

### 3.4. Long-Term L-NAME Treatment Leads to Deteriorated MWM Probe Trial Performance

**Figure 4:**
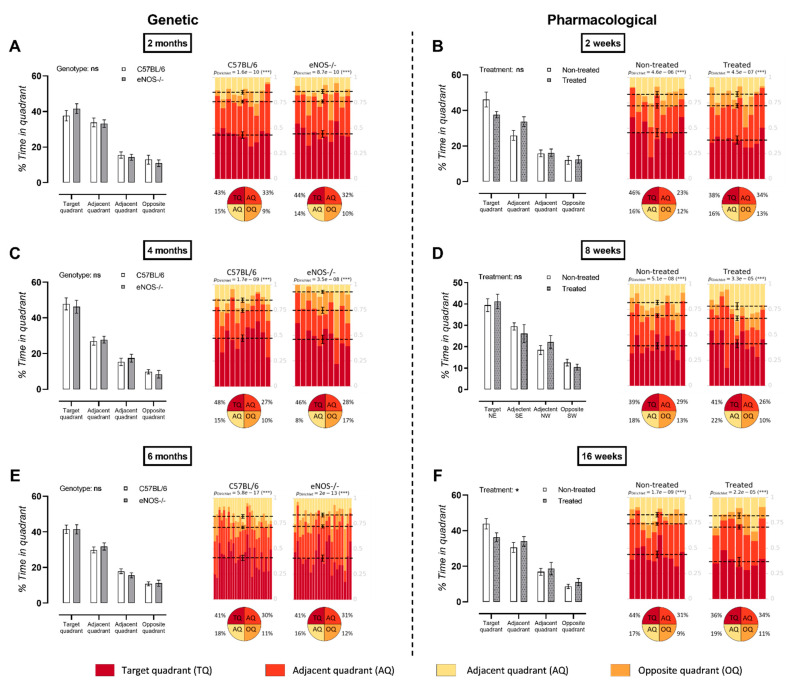
Longitudinal MWM probe trial results. Results for the genetic NO dysfunction murine model are displayed in the left panel with increasing age groups being depicted downward with (**A**) 2 months at the top, (**C**) 4 months in the middle and to (**E**) 6 months at the bottom. Results of the pharmacological NO dysfunction model are shown in the right panel with increasing treatment times being depicted downward from (**B**) 2 weeks at the top, (**D**) 4 weeks in the middle, and (**F**) 16 weeks at the bottom. Factorial ANOVA for the factors genotype/treatment, * *p* < 0.05, *** *p* < 0.002. Data are presented as mean ± SEM. Heatmaps represent statistical analysis via Dirichlet distributions as described earlier [25]. Each column represents the probe trial performance of an individual animal, and each color represents a different quadrant of the MWM. Mean values for the fraction of time spent in each quadrant are represented by a dotted line with respective error bars for the SEM. Average percentages of time spent by the animals in the MWM are indicated in the pie chart beneath the heatmap of each tested group. Respective Dirichlet *p*-values are indicated at the top of each heatmap.

No deterioration of spatial learning or memory probe trial performance was measured in eNOS^−/−^ mice with increasing age (Figure 4A,C,E). In contrast, long-term (26 weeks) but not short-term (2 and 8 weeks) L-NAME treatment led to deterioration in MWM probe trial performance compared to controls (Figure 4B,D,F).

## 4. Discussion

In the present study, we longitudinally induced arterial stiffness in C57BL/6 mice via the inhibition of NO synthases in order to study its effect on cognitive decline. Although NO is a small gaseous signaling molecule with a short half-life of only a few seconds, it has well-defined importance in a large number of physiological processes [7]. There are three NOS isoforms: neuronal NOS (nNOS), inducible NOS (iNOS), and endothelial NOS (eNOS). The nomenclature of these three isoforms reflects their localization of action: nNOS is expressed in neurons, eNOS is expressed in endothelial cells, and iNOS is expressed in glia cells upon brain injury or inflammation. Both nNOS and eNOS produce low levels of NO, while iNOS rapidly produces large amounts of NO [7,9].

Although for many years their functions were thought to be tissue-specific, increasing evidence suggests that they have a broad spectrum of functions. Neuronal NOS is involved in modulating cerebral physiology, such as neurogenesis, learning, memory, and the long-term regulation of synaptic transmission [27,28]. Until recently, eNOS was considered the most prominent NOS isoform in cardiac function. However, a study conducted on left ventricular tissue obtained from explanted human hearts demonstrated the importance of nNOS, rather than eNOS and iNOS, in the pathophysiology of cardiac dysfunction in ischemic heart disease [29]. Additionally, previous research in mice has shown pathological left ventricular remodeling and functional decline after myocardial infarction in nNOS deficient animals [30,31]. Moreover, nNOS is the only NOS isoform that is expressed in intrinsic cardiac neurons and thereby regulates parasympathetic and sympathetic heart rhythm and contractility [32,33]. Here, we reported the presence of hypertrophic cardiomyopathy in L-NAME-treated animals. This contrasted with the cardiac functionality of eNOS^−/−^ mice, where no clear differences were measured compared to C57BL/6 control animals, reinforcing the importance of nNOS rather than eNOS in the functionality of the heart.

In addition, nNOS is involved in the central regulation of blood pressure and plays an important role in the regulation of vascular tone [34,35,36]. In particular, many peripheral smooth muscle tissues, such as those in coronary arteries [37], are innervated by nitrergic nerves containing nNOS activity. From this perspective, both eNOS and nNOS affect the physiological regulation of vascular tone [38]. In the present study, both genetic eNOS knockout and the pharmacological inhibition of nNOS, iNOS, and eNOS by L-NAME resulted in hypertension and elevated aPWVs to a similar extent. As expected, pharmacological treatment showed increasing trends in blood pressure and aPWV with age, while congenital genetic eNOS knockout demonstrated constant trends in blood pressure and aPWV from a young age.

Both mouse models showed markedly increased Eps, with eNOS^−/−^ mice showing more extreme Peterson moduli compared to the aortas of L-NAME-treated animals. This observation can be explained by the genetic loss of eNOS, which ablates NO production in vascular endothelial cells and severely affects the modulation of artery function. In the long term, changes in the lumen shear and wall stress due to abnormal artery modulation could stimulate arterial wall remodeling, including the production and degradation of the extracellular matrix [39,40]. In contrast, the less extreme Eps of L-NAME-treated aortas compared to eNOS^−/−^ aortas may be explained by the rather short duration of treatment and the less invasive elimination of NOS activity by gastrointestinal absorption of the NOS inhibitors via the drinking water.

An evaluation of a progressive decline in hippocampal learning and memory via the MWM test showed a significant effect after 16 weeks of L-NAME treatment. However, the effect of L-NAME treatment fluctuated with time. Specifically, cognitive decline was observed after 2 and 16 weeks of treatment, while an 8 week L-NAME treatment resulted in a similar probe trial performance between the treated and untreated animals. This fluctuation may have been due to the maturation process of the animals; the observed deterioration in probe trial performance after 2 weeks of treatment may have been due to the invasiveness of the treatment for the still-immature animals. Overall, an unmistakable effect of treatment with L-NAME on the cognitive ability of the animals can be observed. These results contrast with ageing eNOS^−/−^ animals that do not show progressive cognitive deterioration until 6 months of age. However, cognitive decline is reported in these animals from the age of 22 months onwards [41,42].

Age is perhaps the most critical factor affecting arterial stiffness and cognition. Our findings clearly demonstrate the age-related presence of arterial stiffness from a young age onward in both mouse models. At the neurobehavioral level, Shoji et. al. recently studied age-related neurobehavioral changes from young adulthood to middle age in male C57BL/6J mice. The authors concluded that 4–6 month old C57BL/6 mice exhibited reduced locomotor activity toward novel environments, depression-related behavior, and deficits in spatial and cued fear memory, amongst others, compared to 2–3 month old C57BL/6 mice [43]. These results align with our recent findings, which demonstrated an age-related cognitive decline in spatial learning and memory in C57BL/6J mice from the age of 6 months onward [44]. In addition to this report, our results showed a significant effect of L-NAME treatment on spatial learning and memory in adult (6 month old) male C57BL/6 mice.

Altogether, our findings demonstrated that the non-selective inhibition of NOS activity induces cardiac dysfunction, arterial stiffness, and a decrease in hippocampal-dependent learning and memory, whereas the genetic deletion of eNOS activity only causes arterial stiffening. Therefore, this result argues for the important role of nNOS in both cardiovascular and neurological pathophysiology and its possible contribution in the convergence between arterial stiffness and cognitive decline. However, since L-NAME has a similar structure to L-arginine, and amino acid transporters are expressed at the blood–brain barrier [45], L-NAME is expected to cross the blood–brain barrier via arginine transporters. L-NAME has a 72% probability of crossing the blood–brain barrier [46], making it conceivable that cognitive decline following L-NAME treatment is merely due to the inhibition of nNOS in the brain, independent of arterial stiffening. However, because a relatively low dose of L-NAME was administered to the mice in this study [16,17], and because L-NAME exhibits the lowest inhibitory constant (Ki) value for nNOS compared to eNOS and iNOS [47,48], it is likely that nNOS activity was inhibited in the brain.

Therefore, more in-depth studies comparing the effect of a selective nNOS inhibitor, e.g., 3-bromo-7-nitroindazole [49,50], with the effect of L-NAME on the brain and the subsequent cerebral NOS expression of both treatments would provide further insight. Further research is also needed to determine the NO-dependent causal relationship between arterial stiffness and cognitive decline, especially in an established model of cognitive decline such as hAPP23+/− transgenic mice. This will allow for further investigation of whether arterial stiffening accelerates or exacerbates pre-existing cognitive decline.

## References

[1] Rabkin S.W. (2012). Arterial stiffness: Detection and consequences in cognitive impairment and dementia of the elderly. J. Alzheimer’s Dis..

[2] van Sloten T.T., Protogerou A.D., Henry R.M., Schram M.T., Launer L.J., Stehouwer C.D. (2015). Association between arterial stiffness, cerebral small vessel disease and cognitive impairment: A systematic review and meta-analysis. Neurosci. Biobehav. Rev..

[3] Iulita M.F., Noriega de la Colina A., Girouard H. (2018). Arterial stiffness, cognitive impairment and dementia: Confounding factor or real risk?. J. Neurochem..

[4] Hendrickx J.O., Martinet W., Van Dam D., De Meyer G.R. (2021). Inflammation, Nitro-Oxidative Stress, Impaired Autophagy, and Insulin Resistance as a Mechanistic Convergence Between Arterial Stiffness and Alzheimer’s Disease. Front. Mol. Biosci..

[5] Bauer V., Sotníková R. (2010). Nitric oxide—The endothelium-derived relaxing factor and its role in endothelial functions. Gen. Physiol. Biophys..

[6] Hasegawa N., Fujie S., Horii N., Miyamoto-Mikami E., Tsuji K., Uchida M., Hamaoka T., Tabata I., Iemitsu M. (2018). Effects of Different Exercise Modes on Arterial Stiffness and Nitric Oxide Synthesis. Med. Sci. Sports Exerc..

[7] Doherty G.H. (2011). Nitric oxide in neurodegeneration: Potential benefits of non-steroidal anti-inflammatories. Neurosci. Bull..

[8] Tewari D., Sah A.N., Bawari S., Nabavi S.F., Dehpour A.R., Shirooie S., Braidy N., Fiebich B.L., Vacca R.A., Nabavi S.M. (2021). Role of Nitric Oxide in Neurodegeneration: Function, Regulation, and Inhibition. Curr. Neuropharmacol..

[9] Liu C., Liang M.C., Soong T.W. (2019). Nitric oxide, iron and neurodegeneration. Front. Neurosci..

[10] Dawson T.M., Dawson V.L. (2018). Nitric oxide signaling in neurodegeneration and cell death. Adv. Pharmacol..

[11] Isabelle M., Simonet S., Ragonnet C., Sansilvestri-Morel P., Clavreul N., Vayssettes-Courchay C., Verbeuren T.J. (2012). Chronic reduction of nitric oxide level in adult spontaneously hypertensive rats induces aortic stiffness similar to old spontaneously hypertensive rats. J. Vasc. Res..

[12] Leloup A.J., Fransen P., Van Hove C.E., Demolder M., De Keulenaer G.W., Schrijvers D.M. (2014). Applanation tonometry in mice: A novel noninvasive technique to assess pulse wave velocity and arterial stiffness. Hypertension.

[13] Baumbach G.L., Sigmund C.D., Faraci F.M. (2004). Structure of cerebral arterioles in mice deficient in expression of the gene for endothelial nitric oxide synthase. Circ. Res..

[14] Faraci F.M., Sigmund C.D., Shesely E.G., Maeda N., Heistad D.D. (1998). Responses of carotid artery in mice deficient in expression of the gene for endothelial NO synthase. Am. J. Physiol.-Heart Circ. Physiol..

[15] Huang Z., Huang P.L., Ma J., Meng W., Ayata C., Fishman M.C., Moskowitz M.A. (1996). Enlarged infarcts in endothelial nitric oxide synthase knockout mice are attenuated by nitro-L-arginine. J. Cereb. Blood Flow Metab..

[16] Nagano K., Ishida J., Unno M., Matsukura T., Fukamizu A. (2013). Apelin elevates blood pressure in ICR mice with L-NAME-induced endothelial dysfunction. Mol. Med. Rep..

[17] Suda O., Tsutsui M., Morishita T., Tanimoto A., Horiuchi M., Tasaki H., Huang P.L., Sasaguri Y., Yanagihara N., Nakashima Y. (2002). Long-term treatment with Nω-nitro-L-arginine methyl ester causes arteriosclerotic coronary lesions in endothelial nitric oxide synthase-deficient mice. Circulation.

[18] Kilkenny C., Browne W., Cuthill I., Emerson M., Altman D.G. (2010). The ARRIVE guidelines. ReqartoCom. PLoS Biol..

[19] Underwood W., Anthony R. (2020). AVMA Guidelines for the Euthanasia of Animals: 2020 Edition.

[20] Van Dam D., D’Hooge R., Staufenbiel M., Van Ginneken C., Van Meir F., De Deyn P.P. (2003). Age-dependent cognitive decline in the APP23 model precedes amyloid deposition. Eur. J. Neurosci..

[21] Van Dam D., Lenders G., De Deyn P.P. (2006). Effect of Morris water maze diameter on visual-spatial learning in different mouse strains. Neurobiol. Learn. Mem..

[22] Feng M., Whitesall S., Zhang Y., Beibel M., Alecy L.D., DiPetrillo K. (2008). Validation of volume–pressure recording tail-cuff blood pressure measurements. Am. J. Hypertens..

[23] Di Lascio N., Stea F., Kusmic C., Sicari R., Faita F. (2014). Non-invasive assessment of pulse wave velocity in mice by means of ultrasound images. Atherosclerosis.

[24] Leloup A.J., Hove C.E., Kurdi A., Moudt S., Martinet W., Meyer G.R., Schrijvers D.M., Keulenaer G.W., Fransen P. (2016). A novel set-up for the ex vivo analysis of mechanical properties of mouse aortic segments stretched at physiological pressure and frequency. J. Physiol..

[25] Maugard M., Doux C., Bonvento G. (2019). A new statistical method to analyze Morris Water Maze data using Dirichlet distribution. F1000Research.

[26] Kluyver T., Ragan-Kelley B., Pérez F., Granger B.E., Bussonnier M., Frederic J., Kelley K., Hamrick J.B., Grout J., Corlay S. Jupyter Notebooks—A Publishing Format for Reproducible Computational Workflows. Proceedings of the 20th International Conference on Electronic Publishing.

[27] Cui J.-M., Li X.-L., Fu F., He J.-P. (2013). Influence of swimming exercise in learning and memory, amino acid content and nNOS expression in prefrontal cortex of aging rats. J. Jilin Univ. (Med. Ed.).

[28] Izumi Y., Clifford D.B., Zorumski C.F. (1992). Inhibition of long-term potentiation by NMDA-mediated nitric oxide release. Science.

[29] Roselló-Lletí E., Carnicer R., Tarazón E., Ortega A., Gil-Cayuela C., Lago F., González-Juanatey J.R., Portolés M., Rivera M. (2016). Human ischemic cardiomyopathy shows cardiac Nos1 translocation and its increased levels are related to left ventricular performance. Sci. Rep..

[30] Saraiva R.M., Minhas K.M., Raju S.V., Barouch L.A., Pitz E., Schuleri K.H., Vandegaer K., Li D., Hare J.M. (2005). Deficiency of neuronal nitric oxide synthase increases mortality and cardiac remodeling after myocardial infarction: Role of nitroso-redox equilibrium. Circulation.

[31] Dawson D., Lygate C.A., Zhang M.-H., Hulbert K., Neubauer S., Casadei B. (2005). nNOS gene deletion exacerbates pathological left ventricular remodeling and functional deterioration after myocardial infarction. Circulation.

[32] Danson E.J., Choate J.K., Paterson D.J. (2005). Cardiac nitric oxide: Emerging role for nNOS in regulating physiological function. Pharmacol. Ther..

[33] Zhang Y.H., Jin C.Z., Jang J.H., Wang Y. (2014). Molecular mechanisms of neuronal nitric oxide synthase in cardiac function and pathophysiology. J. Physiol..

[34] Tandai-Hiruma M., Kato K., Kemuriyama T., Ohta H., Tashiro A., Hagisawa K., Nishida Y. (2013). High blood pressure enhances brain stem neuronal nitric oxide synthase activity in Dahl salt-sensitive rats. Clin. Exp. Pharmacol. Physiol..

[35] Togashi H., Sakuma I., Yoshioka M., Kobayashi T., Yasuda H., Kitabatake A., Saito H., Gross S., Levi R. (1992). A central nervous system action of nitric oxide in blood pressure regulation. J. Pharmacol. Exp. Ther..

[36] Costa E.D., Rezende B.A., Cortes S.F., Lemos V.S. (2016). Neuronal nitric oxide synthase in vascular physiology and diseases. Front. Physiol..

[37] Han G., Ma H., Chintala R., Miyake K., Fulton D.J., Barman S.A., White R.E. (2007). Nongenomic, endothelium-independent effects of estrogen on human coronary smooth muscle are mediated by type I (neuronal) NOS and PI3-kinase-Akt signaling. Am. J. Physiol.-Heart Circ. Physiol..

[38] Melikian N., Seddon M.D., Casadei B., Chowienczyk P.J., Shah A.M. (2009). Neuronal nitric oxide synthase and human vascular regulation. Trends Cardiovasc. Med..

[39] Ward M.R., Pasterkamp G., Yeung A.C., Borst C. (2000). Arterial remodeling: Mechanisms and clinical implications. Circulation.

[40] Humphrey J.D. (2008). Mechanisms of arterial remodeling in hypertension: Coupled roles of wall shear and intramural stress. Hypertension.

[41] Aguirre J., Buttery L., O’Shaughnessy M., Afzal F., de Marticorena I.F., Hukkanen M., Huang P., MacIntyre I., Polak J. (2001). Endothelial nitric oxide synthase gene-deficient mice demonstrate marked retardation in postnatal bone formation, reduced bone volume, and defects in osteoblast maturation and activity. Am. J. Pathol..

[42] Tan X.-L., Xue Y.-Q., Ma T., Wang X., Li J.J., Lan L., Malik K.U., McDonald M.P., Dopico A.M., Liao F.-F. (2015). Partial eNOS deficiency causes spontaneous thrombotic cerebral infarction, amyloid angiopathy and cognitive impairment. Mol. Neurodegener..

[43] Shoji H., Takao K., Hattori S., Miyakawa T. (2016). Age-related changes in behavior in C57BL/6J mice from young adulthood to middle age. Mol. Brain.

[44] Hendrickx J.O., De Moudt S., Calus E., De Deyn P.P., Van Dam D., De Meyer G.R. (2021). Age-related cognitive decline in spatial learning and memory of C57BL/6J mice. Behav. Brain Res..

[45] Montaser A.B., Jarvinen J., Löffler S., Huttunen J., Auriola S., Lehtonen M., Jalkanen A., Huttunen K.M. (2020). L-Type Amino Acid Transporter 1 Enables the Efficient Brain Delivery of Small-Sized Prodrug across the Blood–Brain Barrier and into Human and Mouse Brain Parenchymal Cells. ACS Chem. Neurosci..

[46] Majzúnová M., Pakanová Z., Kvasnička P., Bališ P., Čačányiová S., Dovinová I. (2017). Age-dependent redox status in the brain stem of NO-deficient hypertensive rats. J. Biomed. Sci..

[47] Pfeiffer S., Leopold E., Schmidt K., Brunner F., Mayer B. (1996). Inhibition of nitric oxide synthesis by NG-nitro-L-arginine methyl ester (L-NAME): Requirement for bioactivation to the free acid, NG-nitro-L-arginine. Br. J. Pharmacol..

[48] Reif D.W., McCreedy S.A. (1995). N-nitro-L-arginine and N-monomethyl-L-arginine exhibit a different pattern of inactivation toward the three nitric oxide synthases. Arch. Biochem. Biophys..

[49] Bland-Ward P., Pitcher A., Wallace P., Gaffen Z., Babbedge R., Moore P. (1994). Isoform selectivity of indazole-based nitric oxide synthase inhibitors. Br. J. Pharmacol.-Proc. Suppl..

[50] Bland-Ward P.A., Moore P.K. (1995). 7-Nitro indazole derivatives are potent inhibitors of brain, endothelium and inducible isoforms of nitric oxide synthase. Life Sci..

